# The Potential Impact of Satellite-Retrieved Cloud Parameters on Ground-Level PM_2.5_ Mass and Composition

**DOI:** 10.3390/ijerph14101244

**Published:** 2017-10-18

**Authors:** Jessica H. Belle, Howard H. Chang, Yujie Wang, Xuefei Hu, Alexei Lyapustin, Yang Liu

**Affiliations:** 1 Department of Environmental Health, Emory University, Atlanta, GA 30322, USA; jessica.hartmann.belle@emory.edu (J.H.B.); xuefei.hu@emory.edu (X.H.); 2 Department of Biostatistics and Bioinformatics, Emory University, Atlanta, GA 30322, USA; howard.chang@emory.edu; 3 NASA Goddard Space Flight Center, Greenbelt, MD 20771, USA; yujie.wang-1@nasa.gov (Y.W.); alexei.i.lyapustin@nasa.gov (A.L.)

**Keywords:** PM_2.5_, MAIAC AOD, non-random missingness, cloud properties, RUC/RAP

## Abstract

Satellite-retrieved aerosol optical properties have been extensively used to estimate ground-level fine particulate matter (PM_2.5_) concentrations in support of air pollution health effects research and air quality assessment at the urban to global scales. However, a large proportion, ~70%, of satellite observations of aerosols are missing as a result of cloud-cover, surface brightness, and snow-cover. The resulting PM_2.5_ estimates could therefore be biased due to this non-random data missingness. Cloud-cover in particular has the potential to impact ground-level PM_2.5_ concentrations through complex chemical and physical processes. We developed a series of statistical models using the Multi-Angle Implementation of Atmospheric Correction (MAIAC) aerosol product at 1 km resolution with information from the MODIS cloud product and meteorological information to investigate the extent to which cloud parameters and associated meteorological conditions impact ground-level aerosols at two urban sites in the US: Atlanta and San Francisco. We find that changes in temperature, wind speed, relative humidity, planetary boundary layer height, convective available potential energy, precipitation, cloud effective radius, cloud optical depth, and cloud emissivity are associated with changes in PM_2.5_ concentration and composition, and the changes differ by overpass time and cloud phase as well as between the San Francisco and Atlanta sites. A case-study at the San Francisco site confirmed that accounting for cloud-cover and associated meteorological conditions could substantially alter the spatial distribution of monthly ground-level PM_2.5_ concentrations.

## 1. Introduction

Satellite observations of aerosol optical properties, such as the aerosol optical depth (AOD), are increasingly being used to infer spatial and temporal patterns of fine-mode particulate matter, PM_2.5_, for health studies [1]. However, significant challenges associated with the use of these observations remain. A large proportion of satellite observations are missing (estimated at ~70% in the 10 km AOD products), chiefly as a result of cloud-cover, snow-cover, and surface brightness [2,3]. Previous work to address this gap-filling problem has largely assumed that the observed aerosols are comparable to aerosols that could not be observed [4,5]. Contradicting this assumption, global and US-centric studies have estimated that missing satellite observations result in an underestimation of true PM_2.5_ concentrations, by an average of 20% in the US [6,7]. Additional work has demonstrated that missing satellite data results in over-prediction of ground-level PM_2.5_ concentrations in the summer months and under-prediction in the winter months at higher latitudes [8,9]. More recent work has gone beyond this to examine the contribution of certain drivers, namely the impact of cloud-cover, on PM_2.5_ concentrations at ground level and associated changes in the composition of particulates [10]. The authors found that increased quantities of cloud-cover and increased cloud optical depth were associated with both compositional changes in PM_2.5_ and an overall decrease in concentrations in the southeastern US. These findings suggest that cloud-cover is associated with changes in ground-level PM_2.5_ concentrations and composition. Non-random missingness in satellite retrievals, if not accounted for during exposure estimation of PM_2.5_, can bias health effect estimates in subsequent analyses [11].

Through complex physical and chemical processes, clouds influence the composition, vertical distribution, diurnal patterns, size distribution, and mass concentration of the aerosols beneath them [12]. At the macro scale, clouds are associated with meteorological conditions that govern the micro- and macro-physical properties of both clouds and aerosols, as well as temperature, humidity, wind speed, vertical convection, and planetary boundary layer height [13,14]. All of these can influence particulate concentrations at the ground level by altering rates of deposition, vertical distributions, emissions, and rates of secondary aerosol formation [15]. Relative humidity and temperature additionally interact to influence rates of both cloud and aerosol formation, the properties and phase of the clouds, and gas-particle partitioning of aerosol components [13,15,16,17]. On a more localized scale, clouds, particularly thunderstorms, alter vertical and horizontal convection, block light, and occasionally rain. Changes in convection directly influence vertical distributions of aerosols beneath the cloud, as well as rates of dry deposition [18,19]. Light blockage alters rates of the photochemical reactions responsible for secondary aerosol formation from gaseous precursors in the atmosphere, indirectly altering aerosol composition and concentrations nearer the ground [20,21]. A small fraction of clouds precipitate, in the process depositing airborne aerosols within and beneath the cloud to the ground [22,23]. Near and within the actual cloud, aerosols participate in the process of cloud formation via nucleation scavenging, and can reduce the effective radius of the cloud particles and alter precipitation efficiency [24,25]. Taken together, the result is a complex tangle of interrelationships between clouds, aerosols, and meteorology which results in different aerosol concentrations and composition beneath cloud-cover relative to that observed when the sky is clear.

The combined impact of these processes on ground-level PM_2.5_ has not been directly studied or linked to measurable properties of the clouds themselves. The current study aims to advance our understanding of whether satellite-retrieved cloud properties are associated with changes in ground-level PM_2.5_ concentration and composition, and the extent to which cloud properties are associated with these changes. We examine the empirical relationship between cloud properties and the meteorological conditions associated with cloud presence and ground-level concentrations of PM_2.5_ from area ground monitors over two urban sites in the US: Atlanta and San Francisco, two sites chosen as representative of different aerosol and meteorological regimes. We additionally apply these relationships to account for cloud-cover related missing PM_2.5_ estimates when using AOD to predict ground-level PM_2.5_. We compare results from a model which assumes that the reason for the missing AOD observation is random, to one that accounts specifically for cloud-cover missingness as a distinct phenomenon.

## 2. Materials and Methods

Environmental Protection Agency (EPA) ground observations of 24-h total and speciated PM_2.5_ concentrations between 1 April 2007 and 31 March 2015, were obtained from the EPA’s AirData website [26]. Daily ground observations were used to represent the daily gravimetric mass concentrations at individual stations. Mass reconstruction was used to calculate concentrations of organic carbon (OC), sulfate, and nitrate, elemental carbon (EC), sea salt, and soil to account for unmeasured molecules in the speciation information and ensure that changes in the speciated masses, and model estimates, would be comparable to changes in the matched gravimetric measurements [27]. This aids interpretation by allowing direct comparison of changes in component masses to changes in gravimetric masses. Results are only presented in the paper for the reconstructed OC, sulfate and nitrate mass concentrations. The Chemical Speciation Network (CSN) EC and OC carbon fractions were additionally corrected for differences between Total Optical Transmittance (TOT) and Total Optical Reflectance (TOR) monitors, following previous work [28]. 

**Figure 1:**
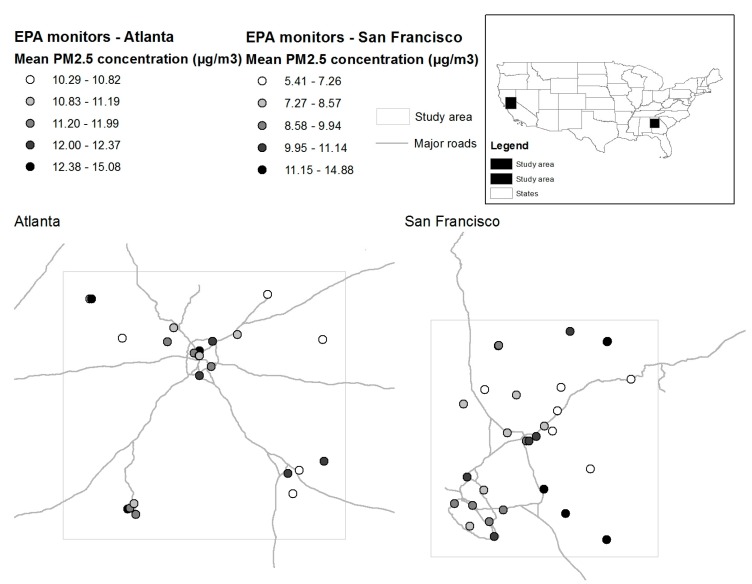
Study site definitions and Environmental Protection Agency (EPA) ground monitor distributions within the two study areas. Mean PM_2.5_ concentrations over the study period are displayed for each monitor.

Monitors located within the study areas surrounding San Francisco and Atlanta, displayed in Figure 1, were collocated with additional data products. The 1 × 1 km twice-daily MAIAC AOD product, with a retrieval accuracy that is comparable to the ±(0.05 + 0.15)*AOD error envelope of the 10 km MODIS AOD products in validation studies, was used to obtain information on AOD and cloud presence/absence, as calculated using the slightly different screening criteria used for aerosol products relative to cloud products [29,30]. The twice-daily MODIS collection 6, daytime cloud product (M*D06) was used to obtain information on cloud emissivity, cloud optical depth (OD), cloud effective radius, and cloud phase [31]. Of these, cloud emissivity, comparable to cloud fraction, and cloud phase are available at 5 km resolution at nadir, while cloud radius and cloud optical depth are available at 1 km resolution at nadir. The 13 × 13 km hourly rapid update cycle (RUC) and its successor the RAPid refresh (RAP) model [32,33] was used to obtain meteorological data on convective available potential energy (CAPE), wind speed, relative humidity (RH), planetary boundary layer (PBL) height, temperature, and precipitation rates in the pixel nearest to each EPA monitoring station during the hours in which twice-daily MODIS pass times from Terra and Aqua occurred. The RUC/RAP meteorological model represents a continuous time-series of moderate resolution assimilated meteorological data, and is known to accurately reproduce vertical profiles of temperature, humidity, and wind speed, all of particular importance to this application [33]. Collocations of satellite and modeled products with EPA observations were processed in a stepwise fashion, starting with MAIAC, so that AOD missingness could be defined separately from its associated climatic conditions and to account for differences in the spatial resolution of each product. First, each 24-h gravimetric EPA observation was matched to the nearest MAIAC pixel within 1 km of the station and defined as AOD missing or present. Using the Quality Assurance (QA) code we further defined each missing AOD value as missing as a result of cloud or other reason, such as snow-cover or fire hot spot. Observations with AOD missing as a result of cloud-cover were then matched to MODIS cloud parameters averaged within a 10 km radius of each EPA observation, and the nearest RUC/RAP observation. Observations where discrepancies existed between the MODIS cloud parameters and the RUC/RAP results on precipitation rate were classified as possibly cloudy, with the remaining cloudy pixels classified according to the cloud phase information from MODIS. This collocation process was repeated separately for both Aqua and Terra MODIS overpasses. Observations were categorized into five categories: definitively uncloudy, possibly cloudy, definitively cloudy but with no phase determination for the cloud, ice clouds, and water clouds. The possibly cloudy and cloudy but of an uncertain phase categories were collapsed in the later analysis into the possibly cloudy category, and definitively uncloudy observations were not analyzed.

In preliminary analyses, a linear mixture modeling approach was used to examine the nature of the relationship between ground-level PM_2.5_ and cloud properties [34,35]. A number of categorical variables were tested as conditioning variables for grouping PM_2.5_ values into sub-populations. The conditional variables included cloud top height, cloud phase, multi-layered cloud flag, the interaction of cloud phase and cloud height and the interaction of multi-layered cloud flag and cloud top height. Of these, the lowest AIC (Akaike information criterion) value was obtained when using cloud phase as the conditioning variable. Since a mixture model with hard separation of components using a categorical variable is statistically very similar to a set of independent models. The final results presented here correspond to simpler, linear mixed effects models run independently for each modeling category.

Specifically, four separate models for the two cloud phases (ice and water), to all observations where AOD was not missing, as well as to all other observations where AOD was missing as a result of possible cloud-cover, were fit to the natural log of the 24-h PM_2.5_ mass concentration at each study location and for each overpass time, making a total of 16 independent models. PM_2.5_ concentrations were log-transformed to normalize the data distribution for these linear models. Results for the possibly cloud models are presented only in the supplementary materials. All models included as predictors RH, wind speed, temperature, PBL height, CAPE, precipitation rate, cloud radius, cloud OD, and cloud emissivity. The model fit to observations where AOD was not missing were fit only to the meteorological parameters RH, wind speed, temperature, PBL height and CAPE. All models additionally included random intercepts for each day of the study period to control for seasonal effects. The equation for this model, used throughout the paper, is given in Equation (1). Here, the natural log of the PM_2.5_ observation at each location (*j*) and time (*i*), is modeled using a random intercept for each day (*β_i_*), and a fixed effect slope (*γ_k_*) for each of k predictors (*X*), plus a random Normal error component (*ε*).

(1) $$\ln\left(PM_{2.5;\;i,j}\right)=Day_{i,j}*\beta_{i}+\sum_{k=1}^{n}X_{i,j,k}*\gamma_{k}\;+\;\varepsilon$$

The same linear mixed effects models (Equation (1)) used to model the impact of cloud cover and meteorological conditions on PM_2.5_ mass were used to model the various PM_2.5_ components, with the goal of identifying the individual component’s relative impacts on the change in total mass. Models were fitted to the natural log of the reconstructed mass of three largest components: sulfate, nitrate, and organic carbon.

We then conducted a case study using a MAIAC AOD-PM model to estimate daily PM_2.5_ where AOD was available. When AOD was not available, values missing in the ungap-filled model were filled in using Equation (3) in the Harvard gap-filling model and were filled in using Equation (1) in the Cloud gap-filling model. We examined differences between the ungap-filled, Harvard gap-filled, and Cloud gap-filled models in the spatial distribution of aerosols from an example monthly estimate choosing January 2012 at the San Francisco site and using a models fit to EPA data over the time period from 2012 to 2014 to predict PM_2.5_. We compared daily predictions made using an ungap-filled model to one that assumes missingness is random (Harvard gap-filled) and to one that assumes cloud-driven missingness (Cloud gap-filled). To accomplish this, the MODIS cloud product and RUC/RAP observations were gridded to the 1 × 1 km MAIAC grid used as the predictive surface for PM_2.5_. The MODIS cloud product was gridded using a method that reconstructs the MODIS polygons using a Voronoi tessellation algorithm from the midpoint locations for each pixel in a granule [3]. These reconstructed polygons were then matched to the MAIAC grid by area to account for the fisheye effect, where pixels towards the edges of the granule are larger than those in the center, still present in the MODIS cloud product. The 1 × 1 km MAIAC grid cells were then matched to the nearest ~13 × 13 km RUC/RAP observation. For pixels where AOD was present a standard prediction model, published in previous works (Equation (2)), was used to predict PM_2.5_ from AOD [36].

(2) $$\begin{array}{cc} PM_{st}=\left(\alpha +u_{t}\right) & +\left({\beta^{\prime }}_{1}+v_{t}\right)AOD_{st}+\left({\beta^{\prime }}_{2k}\right)MetVars_{stk}+{\beta^{\prime }}_{3}Elevation_{s}\\ & +{\beta^{\prime }}_{4}MajorRoads_{s}+{\beta^{\prime }}_{5}ForestCover_{s}+{\beta^{\prime }}_{6}PointEmissions_{s}\\ & +{\varepsilon^{\prime }}_{st}\left(u_{t},\;v_{t},\;w_{t}\right)\sim \;N\left[\left(0,0,0\right),\;\psi \right] \end{array}$$

where MAIAC AOD was absent, Equation (1) was used to impute the missing PM_2.5_ values. We additionally compared results to those obtained over cloudy pixels from an adaptation of the gap-filling model developed by researchers at Harvard, which assumes that all types of missing AOD observations are comparable (Equation (3)) [5,37]. All three models fit a first-stage model to obtain ground-level PM_2.5_ estimates over all times and locations where AOD exists (Equation (2)). In Equation (2), daily PM_2.5_ is modeled using a mixed effects model with fixed (*α*) and daily random intercepts (*u_t_*), fixed (*βʹ*_1_, *βʹ*_2*k*_) and daily random slopes (*v_t_*) for AOD. We additionally included fixed slopes for each of k meteorological variables (MetVars), which included RH, PBL height, temperature, and wind speed as well as fixed slopes (*βʹ*_3–6_) for spatial variables including road length, forest cover percentage, point emissions, and elevation. Equation (2) additionally accounts for error in space and time (*εʹ*_st_(*u_t_*,*v_t_*,*w_t_*)), assuming a multivariate normal distribution centered at 0 *N*[(0,0,0), *ψ*]. The Harvard gap-filled model predicts missing PM_2.5_ via the use of Equation (3), while the gap-filling model utilized in this work accounts for cloud cover by predicting missing PM_2.5_ using Equation (1). Equation (3) predicts the square root of PM_2.5_ concentrations at each location (*s*) and time (*t*), to constrain estimates to be positive, fitting a model with an intercept (*αʹ*), slope for the square root of the daily mean PM_2.5_ concentration over the study area (*βʺ*_1_), and using a spatial smoother (*s*(*X_s_*, *Y_s_*)) fit for each month in the year, predicts the value at each location using the daily mean, assuming random error (*εʺ_st_*). The R statistical computing language was used to fit all models, relying on the packages mgcv, and lme4 [38].

(3) $$\sqrt{PredPM_{st}}=\;\alpha^{\prime }+\;{\beta^{\prime \prime }}_{1}\sqrt{MeanPM_{t}}+s{\left(X_{s},\;Y_{s}\right)}_{k}+\;{\varepsilon^{\prime \prime }}_{st}$$

## 3. Results

### 3.1. Study Area Characteristics

As shown in Table 1 and Figure 1, the Atlanta site contained 23 monitoring sites that collected a total of 26,369 24-h gravimetric observations between 1 April 2007 and 31 March 2015. Study area characteristics for this site are presented in Table 1. Figure 1 shows the spatial distribution of average monitor values. PM_2.5_ concentrations ranged from 2 to 212.5 µg/m^3^, with an average concentration of 11.7 µg/m^3^. Concentration values decreased with time, from an average of 15.9 µg/m^3^ in 2007 to an average of 9.2 µg/m^3^ in 2015, and exhibited seasonal patterns, with higher concentrations in the summer months. Out of the 23 monitoring sites, six additionally collected speciated measurements, which totaled 2410 sets of observations. The largest fraction, both on average and throughout the year, was organic carbon, followed by sulfate.

Of the 26,369 EPA observations, 21,700 could be matched to an Aqua MAIAC pixel, and 21,359 were matched to a Terra MAIAC pixel. Of these, 14,470 (67%) of the Aqua matches and 13,050 (61%) of the Terra matches had a missing AOD observation. For Aqua and Terra the vast majority, were marked as missing due to cloud-cover. This implies that cloud-cover was slightly more common in the mornings in Atlanta. Speciated collocations of monitors and MAIAC pixels followed a similar pattern. Of the 2410 speciated observations, 1997 were matched to an Aqua MAIAC pixel and 1982 to a Terra MAIAC pixel. Of the 1997 matched to Aqua MAIAC, 1313 had AOD missing, while of the 1982 matched to Terra MAAIC, 1192 has AOD missing. For both Aqua and Terra, all observations with missing AOD were marked as missing due to cloud cover.

At the time of the Aqua overpass, the majority (5860) of observations with AOD missing were possibly cloudy, implying disagreement between parameters of products regarding the presence of a cloud in the vicinity of the EPA station. At the time of the Terra overpass, 3556 were marked as possibly cloudy. When cloud presence was definitive at the Aqua overpass time, 4719 were marked as water clouds, 2725 as ice clouds, and 1100 as clouds of an undetermined phase. When cloud presence was definitive at the Terra overpass, 4269 were classified as water clouds, 3210 as ice clouds, and 1994 as clouds of an undetermined phase. Speciated observations followed a similar pattern. These categorizations are additionally presented in Table 2.

As shown in Table 1 and Figure 1, the San Francisco study site contained 28 monitoring stations that recorded a total of 23,357 24-h observations of PM_2.5_ mass concentration over the study period. Concentration values ranged from 2 to 190.2 µg/m^3^ and had a mean value of 9.5 µg/m^3^. Concentrations had no clear trend by year, but varied seasonally from springtime lows of 6 µg/m^3^ to winter highs of 13.6 µg/m^3^. Of the 28 monitoring stations that recorded total mass, 10 additionally recorded speciated mass fractions (2853 sets of observations). The dominant fraction, throughout the year, was organic carbon. In the summer months, this was followed by sulfate, and in the winter months by nitrate.

Of the 23,357 EPA observations in San Francisco, 19,388 were matched to Aqua MAIAC and 19,390 to Terra MAIAC. Nearly 8000 from Aqua and Terra, separately, were missing AOD, 5000 to 6000 fewer than at the Atlanta site. Speciated results followed similar patterns. As can be seen in Table 2, after combination with MODIS cloud and RUC/RAP products, observations within categories of ice cloud, clouds of an uncertain phase, and possibly cloudy were comparable in number to those observed at the Atlanta site. However, water clouds were far fewer in number at the San Francisco site. Similar patterns were observed in the categorization of the speciated results and are presented in Table 2.

### 3.2. Clouds and 24-Hour Gravimetric Mass

Linear mixed effect models relating ground-level PM_2.5_ to meteorological conditions and cloud properties on days and at locations where the MAIAC AOD was missing, separately run for each combination of study site, overpass time, and cloud phase, revealed differences between these categories and dependence of these differences on the cloud phase and the associated variables. As Table 3 demonstrates, cloud-phase specific models outperformed the more ambiguous category containing possible clouds and clouds of an undetermined phase in both sites. Water cloud models also consistently outperformed ice cloud models on this metric, a fact which we ascribe to the fact that the ice cloud category, which includes both thunderstorms and high cirrus clouds, contains a broader range of cloud and meteorological conditions likely to influence aerosol concentrations than the water cloud category, which is more homogenous.

**Figure 2:**
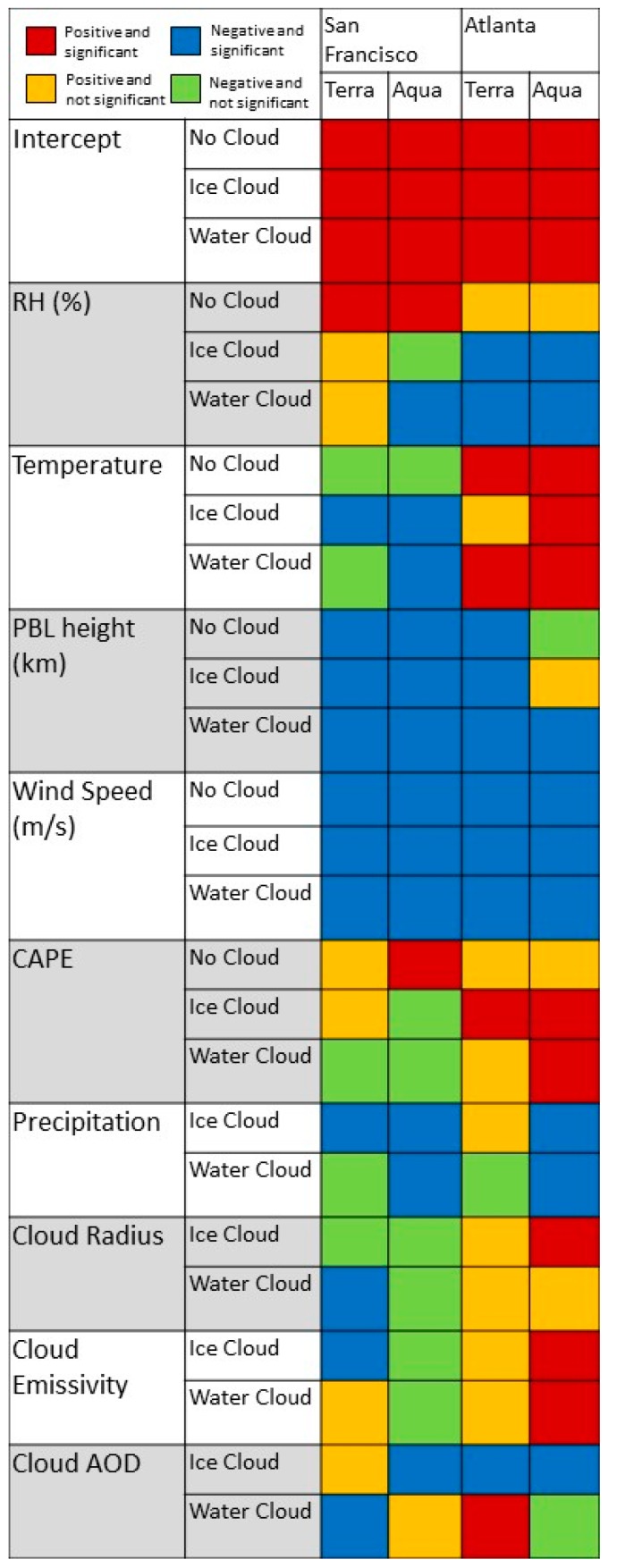
Effect estimate directions and significance for no cloud, ice cloud, and water cloud models. Each estimate is colored according to its direction (positive or negative) and significance (0.05 level). Excepting the intercepts, a positive estimate means an increase in that variable is associated with an increase in PM_2.5_ concentrations, a negative estimate with a decrease in concentrations.

Regression coefficients relating meteorological variables to PM_2.5_ under various cloud conditions are presented in Figure 2 and Supplementary Table S4. With a few exceptions, model results were generally consistent with those from the possibly cloudy and uncloudy observations. Intercepts were all positive, indicating average concentrations in each category that were greater than 1 µg/m^3^. Consistency was also the case for wind speed and PBL height, both of which were associated with decreases in PM_2.5_ concentrations in nearly all models. An increase in RH was associated with a decrease in the PM_2.5_ concentration when clouds were present, with no clear trends by cloud category, site, or overpass. However, in the no cloud model where AOD values were present, RH was associated with an increase in PM_2.5_ concentrations. Higher temperature was associated with a slight increase in ln(PM_2.5_) concentrations in Atlanta, where summertime concentrations tended to be higher, and with a slight decrease in San Francisco, where wintertime concentrations tended to be higher (see Table 1). CAPE, which increases with increasing vertical convection, was strongly negative but not statistically significant at the San Francisco site and slightly positive at the Atlanta site and in the no cloud models at both sites. Precipitation was generally associated with a decrease in ground-level PM_2.5_ at both sites, although this decrease was larger in magnitude at the San Francisco site, where estimates clustered around 0.2 to 0.3, than in Atlanta, where estimates were not significantly different from 0 during the morning overpass. At both sites, precipitation was associated with significant decreases in 24-h PM_2.5_ concentrations when falling in during the afternoon overpass, but with less consistently significant decreases in concentration when falling during the morning overpass.

Cloud properties such as emissivity, radius, and OD, obtained from the MODIS cloud product were also associated with changes in the ground-level PM_2.5_. At the Atlanta site, cloud OD was associated with a significant decrease in PM_2.5_ concentrations when ice clouds were present in the morning and afternoon and with an increase when water clouds were present in the mornings. However, water and ice cloud OD, emissivity, and radius were primarily associated with positive changes in ground-level PM_2.5_ at the Atlanta site. Cloud-cover observed during a MODIS overpass had a more significant and more negative impact on PM_2.5_ concentrations at the San Francisco site. When water clouds were present during the morning overpass, cloud OD was associated with a decrease in ground-level PM_2.5_ concentrations, while increasing cloud radius was associated with a decrease in ground-level PM_2.5_. Cloud emissivity was associated with a decrease in concentration when water clouds were present during the afternoon overpass. Results for ice clouds also differed by overpass at the San Francisco site, although the estimates were comparable, cloud emissivity was a better predictor of concentration changes for morning ice clouds, while cloud OD was a better predictor of concentration changes on the ground for afternoon ice clouds.

### 3.3. Clouds and Speciation of PM_2.5_

Speciated model results are presented in Supplementary Tables S2–S4. An increase in RH was associated with increases in sulfate and nitrate mass at the San Francisco site, with a decrease in nitrate at the Atlanta site, and with decreases in the OC mass at both sites. Increased temperature was associated with an increase in sulfate mass and a decrease in nitrate mass at both sites, and with a decrease in OC mass at the San Francisco site and an increase in OC mass at the Atlanta site. Wind speed was associated with a decrease in mass for all three components, with the exception of sulfate at the San Francisco site. Increases in the PBL height were also associated with decreases in the mass of all components, with one exception for nitrate at the Atlanta site. This same pattern was observed for an increase in CAPE and decreases in component masses with increasing CAPE, with the exception of nitrate in Atlanta. Precipitation was also associated with decreases in component masses for sulfate, nitrate, and OC, particularly when ice clouds were overhead.

Cloud radius was associated with a decrease in the total and sulfate masses at the San Francisco site, but was otherwise not a significant predictor of changes in PM_2.5_ concentrations. Results for sulfate and cloud emissivity or cloud OD at the San Francisco site were mixed, but cloud OD was associated with a decrease in sulfate mass at the Atlanta site. Cloud OD was associated with a decrease in nitrate mass at the San Francisco site and with an increase in nitrate mass at the Atlanta site, although the majority of estimates at the San Francisco site were positive but not significant. Cloud emissivity at the San Francisco site, and Cloud OD at the Atlanta site were associated with increases in OC mass.

### 3.4. Application to MAIAC-Derived PM_2.5_

**Figure 3:**
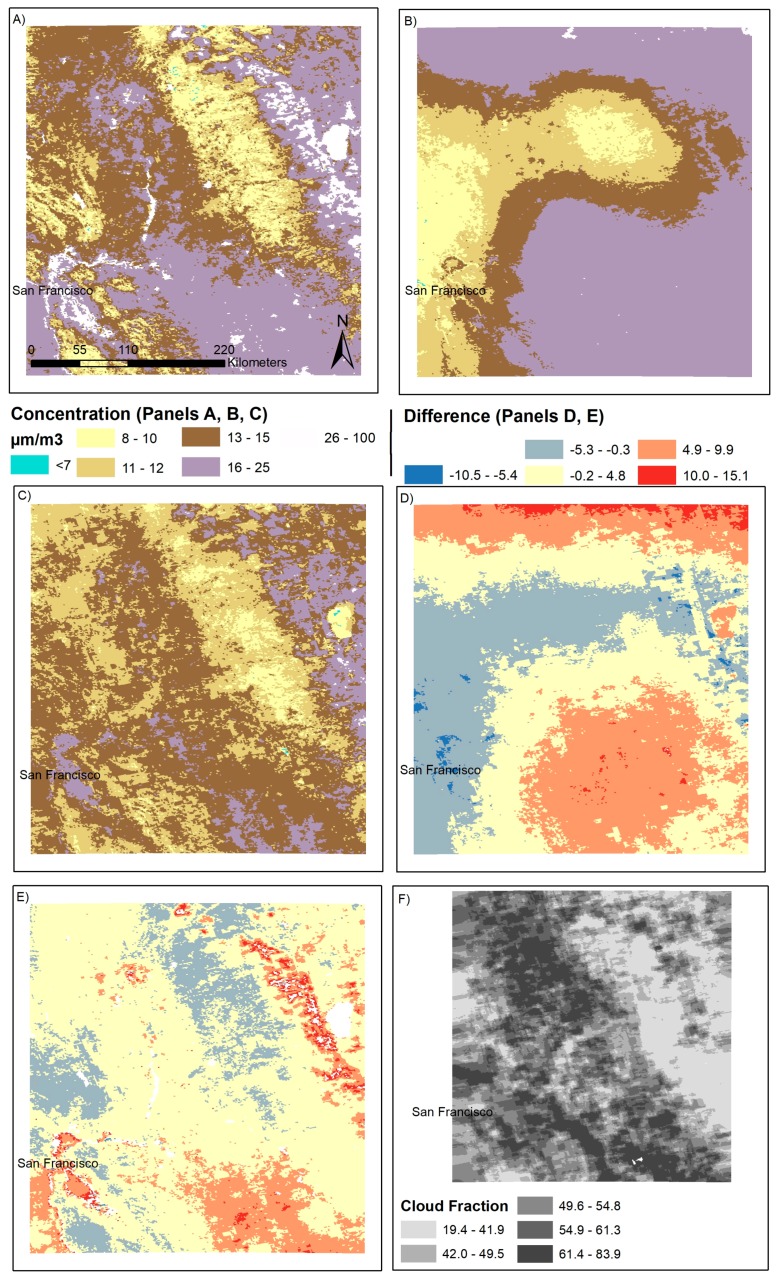
Case study results in San Francisco for January 2012. Results presented are mean concentrations in µg/m^3^ over the month of January for: (**A**) un-gap-filled surface (Equation (2)); (**B**) Harvard model gap-filled surface (Equations (2) and (3)); (**C**) Cloud gap-filled surface (Equations (1) and (2)); (**D**) the difference between the Harvard gap-filled and Cloud gap-filled results at the monthly level; (**E**) the difference between the ungap-filled and Cloud gap-filled results at the monthly level; and (**F**) the fraction of days with a water or ice cloud, as detected by the MODIS M*D06 cloud product.

We applied the cloud model results within the context of a predictive model relating MAIAC AOD to ground-level PM_2.5_ concentrations, comparing results from an ungap-filled AOD to PM_2.5_ model (Equation (2)) with missing observations to those to those from a gap-filling model that ignores cloud-cover, the Harvard gap filling approach (Equation (3)) and a gap-filling model that accounts for cloud properties, the Cloud gap-filling approach (Equation (1)). Results are presented in Figure 3. All three models produce a similar basic spatial pattern for PM_2.5_ concentrations in San Francisco, with higher concentrations in the central valley, lower concentrations over the forested mountains, and higher concentrations on the other side of the mountains near Nevada. However, there are substantial differences in both the monthly averages and spatial patterns between the Harvard and Cloud gap filled results, ranging from −13.14 to 15.52 µg/m^3^ by location. The Harvard gap filled results are considerably smoother than the Cloud gap filled results, also averaging 2.58 µg/m^3^ higher in concentration over the month of January in 2012. It is also worth noting the differences between the non-gap filled and Cloud gap filled results, which average 2.40 µg/m^3^. In Figure 3, the Cloud gap filled monthly average concentrations are lower than the non-gap filled results, particularly over the central valley and metropolitan San Francisco. Cloud fractions (panel E) additionally vary spatially, ranging from 20% to 80%, depending on location.

## 4. Discussion

We examined the relationship between cloud presence and ground-level PM_2.5_ mass and speciation, linking changes in concentration to cloud properties and meteorological conditions. We found that, overall, cloud presence can lead to fairly substantial over or under-prediction of PM_2.5_ concentrations and differences in the spatial patterns of pollutant concentrations when using satellite-observed AOD to estimate ground-level concentrations.

The impact of relative humidity on PM_2.5_ was both negative and consistent between sites, overpass times, and cloud and type. However, results differed by species, with estimates for RH that were negative and largest in magnitude for organic carbon. This implies that most of the changes in total PM_2.5_ mass that were associated with relative humidity result specifically from a decrease in the organic carbon fraction. A likely explanation for this is an increase in the photo-oxidation rates for aromatic hydrocarbons with decreasing humidity [16]. The fact that this association was stronger for organics at the San Francisco site, where NOx concentrations are higher and relative humidity tends to be lower on average, but stronger for gravimetric PM_2.5_ at the Atlanta site, which is known for its high isoprene emissions, also supports this explanation.

The impact of PBL height and the horizontal wind speed on ground-level concentrations of PM_2.5_ were consistently negative, excepting estimates for the association between PBL height and nitrates in Atlanta, implying that increased wind speeds and PBL heights were associated with decreases in PM_2.5_ concentrations. CAPE, an indicator of vertical stability, was more consistently associated with increases in ground-level concentrations of PM_2.5_ on the ground, implying increases with decreasing convective energy, although this association was not consistent. This, in addition to the nitrate results, suggests that future work on this topic should include consideration of vertical convection and distribution of aerosols, as these may also change under cloudy conditions.

Increasing cloud OD, a marker of light blockage from cloud cover, and cloud emissivity, an indicator of the quantity of cloud present, were significantly associated with changes in nitrate, sulfate, and organic carbon concentrations. At both sites, we observed decreases in sulfate and total mass with increasing cloud OD when ice clouds were present. This is consistent with previous results [10] and with an impact specifically from blockage of light to the surface during sunny/fair weather conditions that would otherwise be conducive to the photochemical production of sulfate from gaseous sulfur dioxide [20,39]. Results for water clouds and for nitrate and OC were not consistent between sites, however, and interpretation of these results is less straightforward. This interpretation is further complicated by the fact that cloud-aerosol interactions go both ways, and aerosols have the potential to reduce cloud droplet radii, and thus alter emissivity and OD [21,24]. We had expected to observe an increase in nitrate concentrations with increasing cloud OD or cloud amount, but instead only observed a decrease in nitrate concentrations under afternoon ice clouds in San Francisco. One possible explanation is noise from precipitation events associated with darker cloud-cover that were missing from our precipitation variable. Similarly, we observed an increase in the OC mass with morning water cloud OD at the Atlanta site and emissivity at the San Francisco site. The results point to changes in rates of secondary organic aerosol formation associated with light blockage. Similar to nitrate, recent research points to more rapid, nitrate-driven, nighttime oxidation of isoprene and other volatile organic compounds than through the photo-oxidation routes available during daytime and could explain this increase in concentration with increasing light blockage during the morning hours when nitrate could still be present [20].

Precipitation, via the process of wet deposition, is associated with an overall decrease in PM_2.5_ mass that is larger in magnitude for soluble than for non-soluble PM species [40]. This was observed in our data consistently for ice clouds, which tended to precipitate more, and to some extent for water clouds. The impact of precipitation at the time of the overpass in San Francisco was also larger than that observed in Atlanta. Reasons for this could include the fact that we used a precipitation indicator instead of the precipitation rate, and that it rains more frequently in Atlanta than San Francisco, making the capture of rain during a MODIS overpass time less important relative to 24-h pollutant concentrations.

Finally, we observed a few important differences between sites. Overall, cloud-cover properties and observations at the time of the MODIS overpasses had greater explanatory power in San Francisco than in Atlanta. This was evidenced both by the significance of the cloud OD, cloud emissivity, cloud radius, and precipitation predictors in the models, as well as by the R^2^ values presented in Table 3. The case study included in our results additionally demonstrates that accounting for cloud-cover in a gap-filling model produces differences in monthly results that can be substantial. The observed differences may also stem from the frequency of cloud cover.

We had expected a large proportion of MAIAC retrievals for AOD would be missing, however, a smaller proportion than expected had consistent information on cloud properties between products. Hence, this study was only able to investigate associations for around 50% of the missing AOD observations, limiting the generalizability of conclusions. To mitigate this issue, we have made an effort in the discussion to only highlight results that were consistently observed across the models. However, this also underscores the importance of possible cloud contamination as a source of uncertainty in estimation of ground-level PM_2.5_ from satellite retrievals and is a potentially important area for future research.

## 5. Conclusions

This study demonstrated that clouds are associated with changes in ground-level PM_2.5_ concentration, and these changes are driven by physical and chemical processes associated with cloud cover. We additionally demonstrated that the impact of cloud-driven satellite missingness on our ability to make accurate PM_2.5_ estimates over a surface using this data differs by location. Not accounting for cloud cover and associated meteorological conditions, particularly rainfall, can lead to both over- and under-estimation of PM_2.5_ concentrations. However, additional work is still needed to confirm and clarify the relationships investigated here, particularly into the nature and rationale for the geographic differences observed in these relationships.

Associations between meteorological variables and PM_2.5_ total mass and constituents showed variability across pollutants, cloud types, and locations, but a few important findings stood out. We found that relative humidity is associated with a decrease in the organic component of PM_2.5_ resulting from the humidity dependence of rates of secondary organic aerosol formation. Also, precipitation and changes in rates of secondary aerosol production, indicated by increased cloud OD or cloud emissivity, impact concentration, and speciation of aerosols underneath the clouds.

Our analyses also suggested that not all clouds and locations can be considered equal, and the cloud presence, observed at a specific time of the day, generally matters more in San Francisco than in Atlanta. In San Francisco, we conducted a case study demonstrating changes in spatial patterns of air pollution at the monthly level that were associated with cloud-cover.

## Figures and Tables

**Table 1:** Descriptive results for total gravimetric mass and species fractions.

Study Site and Season		Total Gravimetric Mass			Speciated Mass Fractions			
		No. of Observations	Mean PM_2.5_ (µg/m^3^)	Median PM_2.5_ (µg/m^3^)	No. of Observations	Nitrate *	Sulfate *	Organic Carbon (OC) *
San Francisco	Total	23,357	9.5	7.0	2853	19.7 (2.5)	16.5 (1.3)	47.6 (5.3)
	Winter	6393	13.6	10.2	722	28.5 (5.3)	7.0 (1.0)	52.0 (8.5)
	Spring	5739	6.0	5.5	675	17.9 (1.2)	20.5 (1.3)	42.5 (2.9)
	Summer	5173	8.1	6.5	724	15.2 (1.1)	24.6 (1.6)	43.7 (3.5)
	Fall	6052	9.8	7.9	732	17.4 (2.2)	14.1 (1.3)	51.9 (6.0)
Atlanta	Total	26,369	11.7	10.7	2410	6.7 (0.7)	32.6 (3.4)	46.5 (5.1)
	Winter	6124	10.2	9.2	570	11.9 (1.2)	27.3 (2.6)	48.2 (5.0)
	Spring	6731	11.7	10.6	628	6.8 (0.7)	34.5 (3.6)	45.4 (5.3)
	Summer	6677	13.9	12.8	607	3.3 (0.3)	37.0 (4.4)	43.4 (4.9)
	Fall	6837	10.9	10.2	605	5.3 (0.5)	31.1 (3.1)	49.2 (5.0)

* Values are presented as % total mass (species mass in µg/m^3^).

**Table 2:** Categorization of observations using Multi-Angle Implementation of Atmospheric Correction (MAIAC), then MODIS cloud and rapid update cycle (RUC)/RAPid refresh (RAP) information.

Observation Category		Total Gravimetric Mass				Speciated Mass Fractions			
		Atlanta		San Francisco		Atlanta		San Francisco	
		Aqua	Terra	Aqua	Terra	Aqua	Terra	Aqua	Terra
Matches with MAIAC	All matches	21,700	21,359	19,388	19,390	1997	1982	2385	2394
	Matches with AOD missing	14,470 (67%)	13,050 (61%)	7922 (41%)	7927 (41%)	1313 (66%)	1192 (60%)	868 (36%)	908 (38%)
	Cloud	14,460	13,046	7733	7693	1313	1192	868	908
Including MODIS cloud and RUC/RAP information	Definitively uncloudy	9 (<1%)	2 (<1%)	95 (1%)	178 (2%)	0 (0%)	0 (0%)	0 (0%)	0 (0%)
	Possibly cloudy	5860 (41%)	3556 (27%)	2355 (30%)	4326 (56%)	464 (35%)	480 (40%)	254 (29%)	458 (50%)
	Cloud—uncertain phase	1100 (8%)	1994 (15%)	1124 (15%)	800 (10%)	100 (8%)	141 (12%)	114 (13%)	98 (11%)
	Cloud—Ice cloud	2725 (19%)	3210 (25%)	2397 (31%)	1530 (20%)	286 (22%)	253 (21%)	252 (29%)	204 (22%)
	Cloud—Water cloud	4719 (33%)	4269 (33%)	1929 (25%)	1050 (14%)	459 (35%)	311 (26%)	248 (29%)	147 (16%)

**Table 3:** Model *R*^2^ estimates.

Model *R*^2^ Estimates		Possibly Cloudy	Ice Clouds	Water Clouds
Atlanta	Terra	0.56	0.71	0.74
	Aqua	0.57	0.69	0.73
San Francisco	Terra	0.47	0.60	0.64
	Aqua	0.45	0.54	0.56

## Supplementary Materials

The following are available online at www.mdpi.com/1660-4601/14/10/1244/s1, Table S1. Tabulation of numbers of observations within cloud categories by Environmental Protection Agency (EPA) monitoring site in the Atlanta study area; Table S2. Tabulation of numbers of observations within cloud categories by EPA monitoring site in the San Francisco study area; Table S3. Tabulation of observations within cloud categories by season in Atlanta and San Francisco; Table S4. Model results for gravimetric PM_2.5_ mass concentration. All results are presented as the parameter estimate (standard error) with stars indicating significance if the *p*-value was below 0.05; Table S5. Model results for Sulfate. All results are presented as the parameter estimate (standard error) with stars indicating significance if the *p*-value was below 0.05; Table S6. Model results for Nitrate. All results are presented as the parameter estimate (confidence interval) with stars indicating significance if the *p*-value was below 0.05; Table S7. Model results for organic carbon (OC). All results are presented as the parameter estimate (confidence interval) with stars indicating significance if the *p*-value was below 0.05; Table S8. CV *R*^2^ values from San Francisco case study data over time period from 2012 to 2014.
