# Supplementary material for: The Potential Impact of Satellite-Retrieved Cloud Parameters on Ground-Level PM2.5 Mass and Composition
Source: Int J Environ Res Public Health. 2017 Oct 18;14(10):1244. doi: 10.3390/ijerph14101244 (PMC5664745; doi:10.3390/ijerph14101244)
Supplement: Supplementary file 1 [file ijerph-14-01244-s001.pdf]

Supplementary Materials for “The potential impact of satellite-retrieved cloud parameters on ground-level $PM_{2.5}$ mass and composition”

Table S1. Tabulation of numbers of observations within cloud categories by Environmental Protection Agency (EPA) monitoring site in the Atlanta study area.

State	County FIPS code	Site number	Terra (T) or Aqua (A) overpass	Total # observations with missing AOD	No cloud	Probably cloud	Cloud of undetermined phase	Ice cloud	Water cloud
AL	113	1	A	756	0	73	84	227	372
			T	674	0	435	54	77	108
GA	115	3	A	1225	1	687	80	180	277
			T	1151	0	144	217	360	430
GA	115	5	A	200	0	94	14	28	64
			T	191	0	28	35	44	84
GA	121	32	A	971	0	501	60	140	270
			T	856	0	95	147	225	389
GA	121	39	A	417	0	220	28	57	112
			T	363	0	44	79	118	122
GA	121	48	A	59	0	29	5	11	14
			T	58	0	9	10	12	27
GA	121	56	A	42	0	23	4	7	8
			T	44	0	4	12	19	9
GA	135	2	A	491	0	255	30	68	138
			T	432	0	52	83	135	162
GA	139	3	A	462	1	241	33	65	122
			T	416	1	71	91	123	130
GA	153	1	A	453	0	58	50	136	209
			T	418	0	247	36	55	80
GA	21	12	A	464	0	51	65	132	216
			T	416	0	257	32	54	73
GA	21	7	A	1383	0	148	155	415	665
			T	1295	0	770	97	175	253
GA	215	1	A	481	0	56	47	143	235
			T	409	0	265	36	49	59
GA	215	11	A	473	0	47	57	140	229
			T	419	0	266	33	57	63
GA	215	8	A	314	0	24	25	97	168
			T	271	0	180	26	30	35
GA	223	3	A	510	3	304	28	68	107
			T	453	0	56	79	141	177
GA	319	1	A	474	0	279	25	55	115
			T	425	0	80	61	123	161
GA	59	2	A	452	1	251	24	60	116
			T	402	0	68	67	137	130
GA	63	91	A	520	0	289	31	64	143
			T	466	0	53	81	143	189
GA	67	3	A	1403	0	724	100	216	363
			T	1271	0	126	262	383	500
GA	67	4	A	344	0	201	22	43	78
			T	305	0	51	45	83	126
GA	89	2	A	1555	2	833	86	226	408

			T	1428	1	155	261	434	577
GA	89	2001	A	964	1	472	47	147	297
			T	868	0	100	150	233	385

Table S2. Tabulation of numbers of observations within cloud categories by EPA monitoring site in the San Francisco study area.

State	County FIPS code	Site number	Terra (T) or Aqua (A) overpass	Total # observations with missing AOD	No cloud	Probably cloud	Cloud of undetermined phase	Ice cloud	Water cloud
CA	1	1001	A	45	0	17	6	13	9
			T	48	0	20	4	7	17
CA	1	7	A	287	0	90	41	90	66
			T	268	0	123	32	52	61
CA	1	9	A	39	0	11	9	12	7
			T	47	0	22	4	8	13
CA	101	3	A	823	0	189	127	289	218
			T	810	1	453	80	166	110
CA	11	1002	A	136	0	36	24	38	38
			T	136	0	88	6	29	13
CA	11	7	A	431	0	111	56	158	106
			T	413	0	233	41	107	32
CA	113	1003	A	132	0	30	16	43	43
			T	134	0	73	14	28	19
CA	13	2	A	472	0	146	61	134	131
			T	463	0	233	51	89	90
CA	47	2510	A	227	0	77	29	57	64
			T	233	0	118	28	48	39
CA	57	1001	A	271	43	68	48	70	42
			T	265	67	124	32	30	12
CA	57	5	A	144	2	41	32	40	29
			T	136	4	78	13	28	13
CA	61	3	A	411	1	153	64	128	65
			T	405	2	256	45	83	19
CA	61	6	A	157	0	53	18	40	46
			T	147	0	81	17	29	20
CA	63	1006	A	356	14	98	71	92	81
			T	355	39	210	38	41	27
CA	63	1009	A	307	33	76	53	82	63
			T	281	59	152	28	26	16
CA	63	1010	A	85	0	25	11	31	18
			T	82	4	52	7	18	1
CA	67	10	A	314	0	84	41	100	89
			T	308	0	170	24	64	50
CA	67	4001	A	280	0	67	35	94	84
			T	295	0	173	21	56	45
CA	67	6	A	453	0	118	55	159	121
			T	463	0	265	44	100	54
CA	7	2	A	88	0	24	14	33	17
			T	101	0	64	11	15	11
CA	7	8	A	272	0	70	41	111	50

			T	269	0	159	29	66	15
CA	75	5	A	86	1	30	7	20	28
			T	87	1	42	6	13	25
CA	77	1002	A	657	0	206	78	183	190
			T	662	0	370	62	143	87
CA	81	1001	A	227	0	149	14	28	36
			T	198	0	146	8	13	31
CA	85	5	A	500	0	161	69	137	133
			T	529	0	256	53	117	103
CA	9	1	A	56	1	22	11	14	8
			T	56	1	31	12	5	7
CA	95	4	A	501	0	152	81	162	106
			T	551	0	270	69	121	91
CA	99	5	A	143	0	51	12	39	41
			T	142	0	64	21	28	29

Table S3. Tabulation of observations within cloud categories by season in Atlanta and San Francisco.

		Fall		Winter		Spring		Summer	
		Aqua	Terra	Aqua	Terra	Aqua	Terra	Aqua	Terra
Atlanta	Total missing AOD	3,147	2,787	3,065	2,956	3,779	3,389	4,422	3,899
	No cloud	1	0	8	2	0	0	0	0
	Possible cloud	1,266	672	1,207	738	1,555	929	1,832	1,217
	Cloud of undetermined phase	243	466	365	553	214	365	278	610
	Ice cloud	513	560	778	954	707	913	727	783
	Water cloud	1,124	1,089	707	709	1,303	1,182	1,585	1,289
San Francisco	Total missing AOD	1,862	1,867	3,080	3,130	2,295	2,233	663	654
	No Cloud	5	11	61	109	29	56	0	2
	Possible cloud	564	1,064	829	1,612	700	1,285	262	365
	Cloud of undetermined phase	279	171	458	370	320	195	67	64
	Ice cloud	515	340	1,046	629	670	434	166	127
	Water cloud	499	281	686	410	576	263	168	96

Table S4. Model results for gravimetric $PM_{2.5}$ mass concentration. All results are presented as the parameter estimate (standard error) with stars indicating significance if the p-value was below 0.05].

Atlanta	Aqua	1.97 (1.87, 2.06) *	2.34 (2.21; 2.46) *	2.41 (2.24; 2.58) *	2.52 (2.37; 2.69) *	0.000 (-0.001, 0.001)	-0.004 (-0.006; -0.003) *	-0.004 (-0.006; -0.003) *	-0.007 (-0.009; -0.005) *	0.021 (0.018, 0.024) *	0.016 (0.013; 0.020) *	0.006 (0.002; 0.011) *	0.017 (0.013; 0.020) *	-0.01 (-0.04, 0.03)	-0.04 (-0.08; -0.00) *	0.02 (-0.03; 0.06)	-0.10 (-0.13; -0.06) *	-0.03 (-0.04, -0.03) *	-0.03 (-0.04; -0.02) *	-0.03 (-0.04; -0.02) *	-0.03 (-0.04; -0.02) *	0.02 (-0.00, 0.05)	0.03 (0.01; 0.06) *	0.04 (0.00; 0.07) *	0.05 (0.03; 0.07) *	-0.02 (-0.06; 0.02)	-0.06 (-0.11; -0.02) *	-0.03 (-0.07; -0.00) *	0.000 (-0.001; 0.002)	0.001 (0.000; 0.003) *	0.001 (-0.001; 0.003)	0.000 (0.000; 0.001) *	0.001 (0.000; 0.001) *	0.000 (0.000; 0.001) *	-0.001 (-0.003; 0.001)	-0.001 (-0.002; -0.001) *	-0.000 (-0.001; 0.001)

		San Francisco		
		Terra	Aqua	Terra
Intercept	No Cloud	2.08 (1.96, 2.20) *	2.29 (2.16, 2.41) *	2.06 (1.96, 2.15) *
	Possible Cloud	2.76 (2.57; 2.96) *	3.29 (3.03; 3.55) *	2.70 (2.56; 2.83) *
	Cloud – Ice	2.71 (2.38; 3.05) *	3.05(2.78; 3.33) *	2.63 (2.46; 2.81) *
	Cloud – Water	2.59 (2.16; 3.01) *	3.17 (2.84; 3.49) *	2.50 (2.34; 2.67) *
RH (%)	No Cloud	0.006 (0.004, 0.007) *	0.003 (0.002, 0.004) *	0.00 (-0.001, 0.001)
	Possible Cloud	-0.003 (-0.005; -0.001) *	-0.010 (-0.013; -0.008) *	-0.008 (-0.009; -0.007) *
	Cloud – Ice	0.001 (-0.001; 0.004)	-0.003 (-0.005; 0.000)	-0.005 (-0.007; -0.004) *
	Cloud – Water	0.001 (-0.003; 0.004)	-0.004 (0.007; -0.002) *	-0.006 (-0.007; -0.004) *
Temperature (ºC)	No Cloud	-0.001 (-0.004, 0.002)	-0.000 (-0.003, 0.003)	0.022 (0.020, 0.025) *
	Possible Cloud	-0.016 (-0.021; -0.010) *	-0.017 (-0.023; -0.010) *	0.017 (0.013; 0.020) *
	Cloud – Ice	-0.018 (-0.027; -0.008) *	-0.014 (-0.021; -0.007) *	0.006 (-0.001; 0.010)
	Cloud – Water	-0.006 (-0.017; 0.005)	-0.015 (-0.023; -0.008) *	0.021 (0.018; 0.025) *
PBL height (km)	No Cloud	-0.25 (-0.29, -0.22) *	-0.22 (-0.24, -0.19) *	-0.05 (-0.09, -0.02) *
	Possible Cloud	-0.32 (-0.40; -0.25) *	-0.32 (-0.40; -0.25) *	-0.13 (-0.18; -0.08) *
	Cloud – Ice	-0.22 (-0.33; -0.11) *	-0.27 (-0.35; -0.18) *	-0.11 (-0.17; -0.05) *
	Cloud – Water	-0.41 (-0.54; -0.27) *	-0.31 (-0.39; -0.24) *	-0.17 (-0.21; -0.13) *
Wind Speed (m/s)	No Cloud	-0.08 (-0.09, -0.07) *	-0.08 (-0.09, -0.08) *	-0.04 (-0.04, -0.03) *
	Possible Cloud	-0.10 (-0.11; -0.08) *	-0.11 (-0.13; -0.09) *	-0.04 (-0.05; -0.03) *
	Cloud – Ice	-0.09 (-0.11; -0.06) *	-0.10 (-0.12; -0.08) *	-0.02 (-0.03; -0.01) *
	Cloud – Water	-0.09 (-0.11; -0.07) *	-0.10 (-0.12; -0.08) *	-0.02 (-0.03; -0.01) *
CAPE	No Cloud	0.07 (-0.05, 0.20)	0.11 (0.02, 0.19) *	0.02 (-0.01, 0.04)
	Possible Cloud	-0.26 (-0.54; 0.02)	0.08 (-0.18; 0.33)	0.05 (0.03; 0.08) *
	Cloud – Ice	0.15 (-0.23; 0.54)	-0.06 (-0.37; 0.25)	0.09 (0.05; 0.12) *
	Cloud – Water	-0.37 (-1.25; 0.51)	-0.20 (-0.52; 0.13)	0.02 (-0.05; 0.05)
Precipitation indicator	Possible Cloud	-0.29 (-0.38; -0.21) *	-0.24 (-0.36; -0.12) *	-0.08 (-0.13; -0.04) *
	Cloud – Ice	-0.23 (-0.36; -0.10) *	-0.21 (-0.31; -0.11) *	0.07 (-0.03; 0.05)
	Cloud – Water	-0.06 (-0.22; 0.10)	-0.20 (-0.30; -0.09) *	-0.02 (-0.06; 0.02)
Cloud Radius	Possible Cloud	0.003 (-0.000; 0.006)	0.002 (-0.001; 0.004)	0.001 (0.000; 0.002) *
	Cloud – Ice	-0.001 (-0.004; 0.003)	-0.001 (-0.004; 0.002)	0.000 (-0.001; 0.002)
	Cloud – Water	-0.009 (-0.015; -0.003) *	-0.004 (-0.008; 0.000)	0.000 (-0.003; 0.002)
Cloud emissivity	Possible Cloud	0.001 (-0.000; 0.003)	0.002 (0.000; 0.003) *	0.000 (0.000; 0.001) *
	Cloud – Ice	-0.003 (-0.005; -0.001) *	-0.002 (--0.003; 0.000)	0.000 (-0.001; 0.001)
	Cloud – Water	0.000 (-0.001; 0.002)	-0.001 (-0.003; 0.000)	0.000 (-0.001; 0.000)
Cloud AOD	Possible Cloud	0.000 (-0.002; 0.003)	0.000 (-0.003; 0.003)	0.000 (-0.001; 0.001)
	Cloud – Ice	0.001 (-0.001; 0.004)	-0.004 (-0.006; -0.002) *	-0.002 (-0.002; -0.001) *
	Cloud –Water	-0.003 (-0.005; -0.001) *	0.000 (-0.003; 0.002)	0.001 (0.000; 0.001) *

Table S5. Model results for Sulfate. All results are presented as the parameter estimate (standard error) with stars indicating significance if the p-value was below 0.05].

Atlanta
Aqua
0.07(-0.26, 0.31)
0.54 (0.21; 0.87) *
0.51 (-0.06; 1.07)
0.85 (0.31; 1.39) *
0.013 (0.008, 0.017) *
0.002 (-0.001; 0.006)
0.003 (-0.003; 0.008)
-0.001 (-0.007; 0.005)
0.018 (0.009, 0.028) *
0.017 (0.009; 0.026) *
0.017 (0.002; 0.032) *
0.032 (0.020; 0.044) *
-0.05 (-0.19, 0.10)
0.05 (-0.05; 0.15)
-0.03 (-0.19; 0.13)
-0.11 (-0.25; 0.03)
-0.01 (-0.04, 0.01)
-0.02 (-0.05, 0.01)
-0.02 (-0.06; 0.03)
-0.04 (-0.08; -0.01) *
0.03 (-0.07, 0.14)
0.03 (-0.04; 0.10)
0.01 (-0.10; 0.11)
0.04 (-0.04; 0.11)
-0.18 (-0.30; -0.06) *
-0.14 (-0.32;-0.04)
-0.21 (-0.36; -0.06) *
-0.001 (-0.006; 0.002)
-0.003 (-0.011; 0.004)
-0.010 (-0.020; 0.001)
-0.002 (-0.004; 0.000)
0.001 (-0.002; 0.004)
0.000 (-0.003; 0.002)
0.006 (0.000; 0.012)
-0.005 (-0.007; -0.002) *
-0.002 (-0.006; 0.001)

Table S6. Model results for Nitrate. All results are presented as the parameter estimate (confidence interval) with stars indicating significance if the p-value was below 0.05].

		San Francisco	San Francisco	Atlanta	
		Terra	Aqua	Terra	Aqua
Intercept	No Cloud	1.06 (0.61, 1.50) *	1.30 (0.84, 1.76) *	0.57 (0.28, 0.86) *	0.79 (0.47, 1.12) *
	Possible Cloud	1.65 (0.75; 2.52) *	1.67 (0.69; 2.65) *	1.00 (0.61; 1.38) *	1.01 (0.62; 1.40) *
	Cloud – Ice	1.73 (0.47; 2.99) *	3.86 (2.53; 5.16) *	0.42 (-0.16; 1.01)	0.52 (0.02; 1.01) *
	Cloud – Water	1.32 (-0.25; 2.89)	3.50 (2.25; 4.76) *	1.22 (0.49; 1.90) *	1.11 (0.54; 1.66) *
RH (%)	No Cloud	0.009 (0.005, 0.013) *	[illegible]	[illegible]	[illegible]
	Possible Cloud	0.000 (-0.008; 0.008)	[illegible]	[illegible]	[illegible]
	Cloud – Ice	-0.008 ([illegible])	[illegible]	[illegible]	[illegible]
	Cloud – Water	-0.007 ([illegible])	[illegible]	[illegible]	[illegible]
Temperature (°C)	No Cloud	-0.029 (-0.040, -0.018) *	-0.026 (-0.036, -0.015) *	-0.056 (-0.063, -0.048) *	-0.056 (-0.066, -0.046) *
	Possible Cloud	-0.030 (-0.058; -0.001) *	-0.021 (-0.047; 0.006)	-0.054 (-0.064; -0.043) *	-0.062 (-0.072, -0.051) *
	Cloud – Ice	-0.052 (-0.088; -0.014) *	-0.074 (-0.108; -0.039) *	-0.067 (-0.080; -0.054) *	-0.063 (-0.076; -0.050) *
	Cloud – Water	-0.049 (-0.092; -0.008) *	-0.075 (-0.105; -0.045) *	-0.051 (-0.065; -0.037) *	-0.043 (-0.056; -0.030) *
PBL height (km)	No Cloud	[illegible] (-0.80, -0.48) *	[illegible]	[illegible]	[illegible]
	Possible Cloud	-0.83 ([illegible]) *	[illegible]	[illegible]	[illegible]
	Cloud – Ice	-0.70 ([illegible]) *	[illegible]	[illegible]	[illegible]
	Cloud – Water	-0.80 ([illegible]) *	[illegible]	[illegible]	[illegible]
Wind Speed (m/s)	No Cloud	-0.17 (-0.20, -0.13) *	-0.15 (-0.17, -0.12) *	-0.05 (-0.08, -0.02) *	-0.06 (-0.09, -0.03) *
	Possible Cloud	-0.13 (-0.18; -0.08) *	-0.17 (-0.23; -0.10) *	-0.09 (-0.12; -0.06) *	-0.10 (-0.13; -0.06) *
	Cloud – Ice	-0.10 (-0.17; -0.03) *	-0.12 (-0.18; -0.06) *	-0.01 (-0.06; 0.03)	-0.05 (-0.09; -0.01) *
	Cloud – Water	-0.17 (-0.26; -0.09) *	-0.18 (-0.24; -0.12) *	-0.09 (-0.13; -0.05) *	-0.05 (-0.08; -0.02) *
CAPE	No Cloud	0.02 ([illegible])	[illegible]	[illegible]	[illegible]
	Possible Cloud	-0.77 ([illegible])	[illegible]	[illegible]	[illegible]
	Cloud – Ice	-3.01 ([illegible])	[illegible]	[illegible]	[illegible]
	Cloud – Water	1.38 ([illegible])	[illegible]	[illegible]	[illegible]
Precipitation indicator	Possible Cloud	-0.35 (-0.68; -0.02) *	-0.53 (-1.03; -0.03) *	-0.20 (-0.34; -0.05) *	-0.13 (-0.27; 0.02)
	Cloud – Ice	-1.04 (-1.55; -0.51) *	-0.60 (-1.03; -0.18) *	-0.33 (-0.52; -0.15) *	-0.27 (-0.44; -0.10) *
	Cloud – Water	-0.33 (-0.94; 0.30)	-0.19 (-0.53; 0.16)	0.12 (-0.10; 0.34)	-0.12 (-0.28; 0.04)
Cloud Radius	Possible Cloud	[illegible]	[illegible]	[illegible]	[illegible]
	Cloud – Ice	0.007 ([illegible])	[illegible]	[illegible]	[illegible]
	Cloud – Water	-0.007 ([illegible])	[illegible]	[illegible]	[illegible]
Cloud emissivity	Possible Cloud	0.001 (-0.003; 0.005)	-0.003 (-0.007; 0.001)	-0.001 (-0.003; 0.001)	0.000 (-0.003; 0.002)
	Cloud – Ice	-0.004 (-0.009; 0.001)	-0.005 (--0.010; 0.001)	-0.002 (-0.006; 0.001)	-0.001 (-0.004; 0.002)
	Cloud – Water	0.002 (-0.004; 0.007)	-0.004 (-0.007; -0.000) *	-0.001 (-0.003; 0.002)	-0.002 (-0.004; 0.000)
Cloud AOD	Possible Cloud	[illegible]	[illegible]	[illegible]	[illegible]
	Cloud – Ice	0.010 ([illegible]) *	[illegible]	[illegible]	[illegible]
	Cloud –Water	0.004 ([illegible])	[illegible]	[illegible]	[illegible]

Table S7. Model results for organic carbon (OC). All results are presented as the parameter estimate (confidence interval) with stars indicating significance if the p-value was below 0.05].

Table S8. CV R^2 values from San Francisco case study data over time period from 2012–2014.

	No gap filling	Harvard gap filling	Cloud gap filling

		San Francisco		Atlanta	
		Terra	Aqua	Terra	Aqua
Intercept	No Cloud	4.15 (3.75, 4.55) *	3.96 (3.54, 4.38) *	1.87 (1.60, 2.15) *	1.71 (1.43, 2.00) *
	Possible Cloud	3.55 (2.85; 4.24) *	3.38 (2.63; 4.13) *	2.72 (2.37; 3.07) *	1.95 (1.57; 2.32) *
	Cloud – Ice	4.45 (3.34; 5.58) *	5.02 (3.82; 6.22) *	2.38 (1.80; 2.96) *	2.08 (1.61; 2.54) *
	Cloud – Water	4.80 (2.78; 6.88) *	5.15 (3.62; 6.78) *	2.74 (2.19; 3.28) *	2.53 (2.03; 3.00) *
RH (%)	No Cloud	-0.019 (-0.023, -0.016) *	-0.020 (-0.024, 00.016) *	-0.004 (-0.008, -0.001) *	-0.009 (-0.013, -0.005) *
	Possible Cloud	-0.015 (-0.020; -0.010) *	-0.016 (-0.022; -0.010) *	-0.015 (-0.019; -0.012) *	-0.009 (-0.013; -0.005) *
	Cloud – Ice	-0.016 (-0.027; -0.007) *	-0.021 (-0.031; -0.011) *	-0.010 (-0.015; -0.004) *	-0.008 (-0.013; -0.004) *
	Cloud – Water	-0.016 (-0.034; 0.002)	-0.024 (-0.036; -0.013) *	-0.019 (-0.024; -0.014) *	-0.016 (-0.020; -0.011) *
Temperature (ºC)	No Cloud	-0.053 (-0.062, -0.043) *	-0.048 (-0.057, -0.038) *	0.016 (0.009, 0.023) *	0.024 (0.016, 0.032) *
	Possible Cloud	-0.044 (-0.065; -0.023) *	-0.025 (-0.045; -0.003) *	0.013 (0.004; 0.022) *	0.017 (0.008; 0.028) *
	Cloud – Ice	-0.077 (-0.108; -0.046) *	-0.081 (-0.112; -0.050) *	0.011 (-0.002; 0.024)	0.015 (0.003; 0.027) *
	Cloud – Water	-0.093 (-0.145; -0.040) *	-0.078 (-0.113; -0.044) *	0.031 (0.020; 0.041) *	0.014 (0.003; 0.025) *
PBL height (km)	No Cloud	-0.76 (-0.90, -0.61) *	-0.39 (-0.50, -0.29) *	-0.16 (-0.28, -0.04) *	-0.10 (-0.23, 0.03)
	Possible Cloud	-0.75 (-0.95; -0.55) *	-0.49 (-0.66; -0.32) *	-0.23 (-0.36; -0.10) *	-0.08 (-0.21; 0.03)
	Cloud – Ice	-0.69 (-1.10; -0.28) *	-0.73 (-1.04; -0.42) *	-0.16 (-0.38; 0.04)	-0.03 (-0.17; 0.11)
	Cloud – Water	-0.85 (-1.41; -0.30) *	-0.93 (-1.27; -0.61) *	-0.43 (-0.57; -0.29) *	-0.14 (-0.26; -0.02) *
Wind Speed (m/s)	No Cloud	-0.18 (-0.21, -0.15) *	-0.15 (-0.18, -0.13) *	-0.12 (-0.14, -0.09) *	-0.08 (-0.11, -0.05) *
	Possible Cloud	-0.12 (-0.16; -0.08) *	-0.15 (-0.19; -0.11) *	-0.13 (-0.16; -0.10) *	-0.11 (-0.15; -0.08) *
	Cloud – Ice	-0.15 (-0.23; -0.08) *	-0.14 (-0.20; -0.09) *	-0.09 (-0.13; -0.04) *	-0.11 (-0.15; -0.07) *
	Cloud – Water	-0.23 (-0.36; -0.12) *	-0.21 (-0.28; -0.14) *	-0.09 (-0.12; -0.06) *	-0.08 (-0.11; -0.05) *
CAPE	No Cloud	1.26 (0.65, 1.87) *	0.86 (0.46, 1.27) *	0.03 (-0.04, 0.11)	-0.01 (-0.10, 0.08)
	Possible Cloud	-0.48 (-1.60; 0.64)	0.46 (-0.48; 1.41)	0.02 (-0.03; 0.08)	-0.03 (-0.09; 0.04)
	Cloud – Ice	0.34 (-2.04; 2.87)	-1.07 (-3.14; 1.04)	0.00 (-0.10; 0.09)	-0.08 (-0.16; 0.00)
	Cloud – Water	2.58 (-1.99; 7.26)	0.11 (-1.45; 1.68)	-0.04 (-0.12; 0.04)	0.07 (0.01; 0.13) *
Precipitation indicator	Possible Cloud	-0.23 (-0.49; -0.03)	-0.13 (-0.47; 0.21)	-0.09 (-0.23; 0.04)	-0.11 (-0.26; 0.04)
	Cloud – Ice	-0.35 (-0.86; -0.17)	-0.11 (-0.50; 0.28)	-0.23 (-0.40; -0.06) *	-0.19 (-0.35; -0.03) *
	Cloud – Water	-0.48 (-0.33; 1.31)	-0.02 (-0.42; 0.39)	0.21 (0.04; 0.38) *	-0.06 (-0.19; 0.08)
Cloud Radius	Possible Cloud	0.003 (-0.003; 0.010)	0.004 (-0.001; 0.009)	0.000 (-0.006; 0.005)	0.004 (-0.002; 0.011)
	Cloud – Ice	-0.001 (-0.012; 0.009)	-0.001 (-0.011; 0.009)	-0.002 (-0.008; 0.005)	-0.002 (-0.009; 0.005)
	Cloud – Water	-0.008 (-0.030; 0.015)	-0.013 (-0.029; 0.003)	-0.003 (-0.012; 0.005)	0.001 (-0.008; 0.010)
Cloud emissivity	Possible Cloud	0.002 (-0.001; 0.005)	-0.004 (-0.007; -0.002) *	-0.002 (-0.004; 0.000)	-0.001 (-0.003; 0.002)
	Cloud – Ice	-0.002 (-0.006; -0.003)	-0.002 (-0.006; 0.003)	0.000 (-0.003; 0.003)	0.001 (-0.002; 0.004)
	Cloud – Water	-0.002 (-0.009; 0.005)	0.002 (-0.002; 0.006)	-0.001 (-0.003; 0.000)	-0.002 (-0.004; -0.000) *
Cloud AOD	Possible Cloud	-0.003 (-0.010; 0.003)	0.004 (-0.002; 0.011)	0.005 (-0.001; 0.011)	0.006 (-0.002; 0.014)
	Cloud – Ice	0.002 (-0.005; 0.010)	-0.007 (-0.016; 0.001)	-0.003 (-0.007; 0.001)	-0.002 (-0.005; 0.001)
	Cloud –Water	0.001 (-0.011; -0.014)	-0.001 (-0.009; 0.007)	0.004 (0.000; 0.007) *	0.001 (-0.002; 0.004)

R^2 value	0.32	0.29	0.33
